# In regard to Minniti et al.: Current status and recent advances in resection cavity irradiation of brain metastases—roundup to cover all angles

**DOI:** 10.1186/s13014-021-01854-x

**Published:** 2021-07-10

**Authors:** Gustavo R. Sarria, Christopher P. Cifarelli, Henning Kahl, Frank A. Giordano

**Affiliations:** 1 Department of Radiation Oncology, University Hospital Bonn, University of Bonn, Venusberg Campus 1 Building 55, 53127 Bonn, Germany; 2 Department of Neurosurgery and Radiation Oncology, West Virginia University, Morgantown, WV USA; 3 Department of Radiation Oncology, University Hospital Augsburg, Augsburg, Germany

**Keywords:** Brain metastases, Intraoperative radiotherapy, Brachytherapy, Kilovoltage

## Abstract

We read with great interest the recent review, entitled “Current status and recent advances in resection cavity irradiation of brain metastases”. It is a comprehensive summary of currently available techniques for treatment of post-resection cavity in patients with this diagnosis. We would like to complement this manuscript by including intraoperative techniques as other viable approaches in the management of these patients.

## Letter body

In regard of the recently published review by *Minniti *et al*.* [1]*,* which summarizes the current evidence on resection cavity irradiation of brain metastases (BM), we would like to provide our insights of other methods for this purpose. The development of intraoperative radiotherapy (IORT) over the past four decades has opened a vast spectrum of possible applications for malignancy treatment. Since the first published experiences from Japan in the early 1980’s on treating gliomas and BM, employing an electron-based IORT device (IOERT), timely resurgence of this technique has paved its road to evolving into a modern therapeutic alternative for BM. In addition, a growing body of evidence for intraoperative brachytherapy further contributes to widen the therapeutic arsenal in this setting.

Although no large randomized trials are available, both historical and current evidence in the management of BM with brachytherapy implants support this irradiation method. Reinforcing historical reports from over 25 years ago [2], a more recent prospective study demonstrated the feasibility and safety of applying Cs^131^ intraoperatively to the resection cavity in 42 patients carrying 46 BM, prescribing 80 Gy at 5 mm depth, reaching a local control rate of 100% after 1.5 years with no cases of radionecrosis (RN) [3]. Successive retrospective analysis have suggested similar results, with a relatively low rate of RN [4, 5]. However, evidence from these rather small cohorts should be carefully interpreted.

The dawn of IORT is directly related to low-energy x-rays (kilovoltage-kV), initially published as early as 1907 for treatment of abdominal malignancies [6]. Modern technology has adapted kV units to portable and versatile devices. Their generally low shielding requirements turns them of use for in- and outpatient management. Under the general principle of high-conformal dose distribution and healthy tissue sparing, this sharp fall-off dose of 50-kV accelerators enables concentrating increased doses on a quite well defined target area. Treatment application through a spherical applicator allows a homogeneous delivery doses in a circumferential fashion, providing excellent coverage of the entire resection cavity and a steep dose drop to approximately 30% of the surface dose at 1 cm [7]. Nevertheless, it must be noted that thorough patient selection should be performed, in order to determine applicability, proximity to organs at risk (OAR) and dose to deliver. Furthermore, a final decision might be made only during resection, after live assessment under direct view.

An early IOERT report with a considerable number of patients with BM including 43 metastatic lesions treated with a mean 19.9 Gy (18–25) dose prescribed at 1-cm depth, resulted in seven patients developing local failure within 1 year after treatment. Being approximately an 84% rate, these results remain comparable to those resection plus whole-brain radiotherapy (WBRT) or stereotactic radiosurgery (SRS) approaches [8]. Nonetheless, elevated rates of “encephalic atrophy” and “dementia” were described, likely due to the large healthy-tissue volume exposed to the prescription dose [9].

Dosimetric comparisons have determined the feasibility of dose escalation with kilovoltage-IORT (kV-IORT), confirming a clear benefit in terms of healthy tissue sparing [10]. After these considerations, initial clinical experiences with kV-IORT have been reported during the last decade. A recent multi-institutional retrospective study by our group reported on 54 patients who received superficial doses of mostly 20–30 Gy, with 1-year local control and overall survival rates of 88% and 73%, respectively. It is noteworthy to point that treatment times varied between 12.1 to 22.3 min, according to the applicator diameter [11]. Ongoing prospective trials (NCT03226483, NCT04690348) and the initiation of an international kV-IORT registry will deliver data on the future value of these technologies in the treatment of brain metastases.

## Data Availability

Not applicable.

## Abbreviations

BM: Brain metastases
IORT: Intraoperative radiotherapy
IOERT: Intraoperative electron radiotherapy
RN: Radionecrosis
kV: Kilovoltage
OAR: Organs at risk
WBRT: Whole-brain radiotherapy
